# *Eurosurveillance* reviewers in 2017

**DOI:** 10.2807/1560-7917.ES.2018.23.1.180104-1

**Published:** 2018-01-04

**Authors:** Eurosurveillance editorial team

**Affiliations:** 1 European Centre for Disease Prevention and Control (ECDC), Stockholm, Sweden

The editorial team is grateful to all the experts who dedicated their time to peer-review our articles over the past 12 months. We wish to thank them and all those whose names are not listed below but gave helpful advice and support. We apologise to any reviewers whose names we may have missed.

Our reviewers in 2017 were:

Frank Aarestrup; Preben Aavitsland; Muna Abu Sin; Ibrahim Abubakar; Natalie Adams; Cornelia Adlhoch; Jan Albert; Barbara Albiger; Franz Allerberger; Gayatri Amirthalingam; Nick Andrews; Denise Antona; Katherine Atkinson; Sabrina Bacci; Michael Baker; Elizabeth Bancroft; Francis Barin; Ian Barr; Aldert Bart; Cornelius Bartels; Luisa Barzon; Leonardo Basco; Anna Baumann-Popczyk; Frank Beard; Angela Bechini; Ermias Belay; Rutger Bennet; Kimberley Benschop; Anne Berger-Carbonne; Silvia Bertagnolio; Marianne Besnard; Rijkelt Beumer; Philippe Beutels; Beverly Biggs; Zeno Bisoffi; Carina Blackmore; Jesse Blanton; Pierre-Yves Boelle; Shelly Bolotin; Magnus Boman; Marc Bonten; Thijs Bosch; Arnold Bosman; Herve Bourhy; Virginia Bowen; Karoline Bragstad; Christian Brandt; Andrew Breed; Viviane Bremer; Eeva Broberg; Udo Buchholz; Jane Buxton; Stefan Börjesson; Sindy Böttcher; Saverio Caini; Helen Campbell; Christine Campese; Yehuda Carmeli; João André Carriço; Jordi Casabona; Jean-Sebastien Casalegno; Francesco Castelli; Jesus Castilla Catalan; Dominique Caugant; Rachel Chalmers; Mark Chen; Stephane Chevaliez; Gerardo Chowell; Peer Brehm Christensen; Abrar Chughtai; Konstantin Chumakov; Nadia Ciampa; Bruno Ciancio; Vittoria Colizza; Simon Collin; Geoffrey Coombs; Helena Cortes Martins; Laurent Cotte; Suzanne Cotter; Anthony Cousien; Susan Cowan; Benjamin Cowling; Ingmar Cox; Adam Crawley; Natasha Crowcroft; Ida Czumbel; Viktor Dahl; Cédric Dananché; Niklas Danielsson; Mark Danta; Siddhartha Sankar Datta; Jean-Philippe David; Nadav Davidovitch; Pieter de Boer; Jesús de Pedro Cuesta; Gaston De Serres; Rory D. de Vries; Sylvia Declich; Valerie Delpech; Tarik Derrough; Sarika Desai; Sergio Di Martino; Liselotte Diaz Högberg; Sabine Diedrich; Mercedes Diez; Gerhard Dobler; Yohei Doi; Dragoslav Domanovic; Lisa Domegan; Raquel Duarte; Sandra Dudareva-Vizule; Erika Duffell; Erwin Duizer; Alexandre Duvignaud; Michael Edelstein; Ronald E Engle; Connie Erkens; Douglas Esposito; Steen Ethelberg; Matthieu Eveillard; Mirko Faber; Michael Farrell; Margaret Fearon; Jill Ferdinands; Laura Fernàndez-López; Antonietta Filia; William Fischer; Thea Fischer; Marc Fischer; David Fisman; Claude Flamand; Brendan Flannery; Laure Fonteneau; Frode Forland; Ron Fouchier; Pieter Fraaij; Ellen Fragaszy; Christina Frank; Eelco Franz; Joanne Freedman; Alexander Friedrich; Ingrid Friesema; Angelika Fruth; Alicia Fry; Giovanni Gabutti; Kartini Gadroen; Monica Galiano; Lauren Gardner; Patricia Garvey; Maja George; Peter Gerner-Smidt; Giovanni Gherardi; Eleni Giamarellou; Monica Gianfranceschi; Heather Gidding; Nicolas Gilbert; Vladimir Gilca; Enrico Girardi; Anja Globig; Pere Godoy; Daniel Golparian; Claire Gordon; Marga Goris; Magnus Gottfredsson; Luigi Gradoni; Tine Grammens; Kathie Grant; Stephen Graves; Michel Grignon; Diane Gross; Andrew Grulich; Beatriz Guerra; Barbara Gunsenheimer-Bartmeyer; Jean-Paul Guthmann; Huldrych Günthard; Walter Haas; Josipa Habus; Ágnes Hajdu; Irene Hall; Ian Hall; Anette Hammerum; Dag Harmsen; Michael Hartal; Heli Harvala; Henrik Hasman; Angelos Hatzakis; Barbara Hauer; Amber Haynes; Shirin Heidari; Franz Heinz; Wiebke Hellenbrand; Rene Hendriksen; Ursel Heudorf; Peter Hoffman; Sven Hoffner; Vahur Hollo; Judit Krisztina Horvath; Jaroslav Hrabak; Darko Hren; Gwenda Hughes; Benedikt Huttner; Judith Hübschen; Thomas Inns; Angela Iuliano; Jacques Izopet; Michael Jackson; Katri Jalava; Klaus Jansen; Adam Jenney; Kari Johansen; Nathalie Jourdan-da Silva; Laila Kahwati; Bernard Kaic; Rolf Kaiser; Tommi Karki; Margaret Kay; Brigitte Keller-Stanislawski; Kamran Khan; Yury Khudyakov; Pete Kinross; Hilary Kirkbride; Peter Kirwan; Esther Kissling; Anda Kivite; R. Monina Klevens; Hilde Kløvstad; Gary Kobinger; Anke Kohlenberg; Frank Konings; Marion Koopmans; Michael Kosoy; Gerard Krause; Peter Kreidl; Karl G. Kristinsson; Paul Krogstad; Ed Kuijper; Jeffrey Kwong; Angie Lackenby; Shamez Ladhani; Kaja-Triin Laisaar; Marlyn Langford; Simone Lanini; Amparo Larrauri; Jesper Larsen; Guiseppe Latorre; Camille Lebarbenchon; Tinne Lernout; Jessica Leung; Alexandra Levitt; Tore Lier; Marshall Lightowlers; Giedrius Likatavicius; Jing Liu-Helmersson; Luis Lowe; Jay Lucidarme; Outi Lyytikäinen; Daniel Lévy-Bruhl; Knut Lönnroth; Ian Mackay; Fabio Magurano; Suman Majumdar; Helena Maltezou; Ula Maniewski; Jean-Michel Mansuy; Ulrich Marcus; Laurence Marrama; Kim Marsh; Rebecca Martin; Emily Martin; Valentina Marziano; Brian Maskery; Andrew McArthur; Jim McMenamin; Adam Meijer; Angeliki Melidou; Tanya Melillo Fenech; Alexander Mellmann; Kassiani Mellou; Stefano Merler; Tony Merritt; Thomas Mertens; Thomas Mollet; Dominique Monnet; Arnold Monto; Hannah Moore; Orna Mor; Jacob Moran-Gilad; Serge Morand; Alain Moren; Jane Morgan; Joël Mossong; Antons Mozalevskis; Viktor Mravčík; Lapo Mughini-Gras; Mark Muscat; James Musser; Didier Musso; Mette Myrmel; Kåre Mølbak; Thomas Müller; Celine A Nadon; Andreas Neumayr; Emanuele Nicastri; Matthias Niedrig; Eva Møller Nielsen; Sandra Niendorf; Hubert Niesters; Taina Niskanen; Harold Noel; Hanne Nokleby; Teymur Noori; Helene Norder; Baltazar Nunes; Pat Nuttall; Domingo Nuñez; Darina O'Flanagan; Ruth Offergeld; Makoto Onishi; Laura Pabst; Daniel Palm; Takis Panagiotopoulos; Marcus Panning; Annalisa Pantosti; Dimitrios Paraskevis; Elena Pariani; R David Parker; Suzan Pas; Richard Pebody; Malik Peiris; Joseph Peiris; Luisa Peixe; Pasi Penttinen; Agnès Perrin; Martin Petric; Mariska Petrignani; Günter Pfaff; Dina Pfeifer; Yvonne Pfeifer; Anastasia Pharris; Denis Pierard; Sylvie Pillet; Rosa Pintó; Diamantis Plachouras; Laurent Poirel; Isabelle Poujol; Edoardo Pozio; Elisabeth Presterl; Jan Prins; Matthias Pulz; Mikkel Quam; Lul Raka; Brian Raphael; Lasse Dam Rasmussen; Helena Rebelo-de-Andrade; Claudia Reinheimer; Chantal Reusken; Giovanni Rezza; Anders Rhod Larsen; Caterina Rizzo; Emmanuel Robesyn; Mohammad Rokni; Roberto Romi; Karin Ronning; Anne-Marie Roque-Afonso; Senia Rosales-Klintz; Mirko Rossi; Gian Maria Rossolini; Omar Rota Stabelli; Adam Roth; Etienne Ruppe; Kate Russell; Malgorzata Sadkowska-Todys; Daniel Sagebiel; Mika Salminen; Lamia Samad; Jussi Sane; Andre Sasse; Rachel Savage; Aaron Scherer; Barbara Schimmer; Jonas Schmidt-Chanasit; Holger Scholz; Jennifer Schuster; Brunhilde Schweiger; Tara Sealy; Harald Seifert; Torsten Semmler; Helena Seth-Smith; Torsten Seuberlich; Ettore Severi; Otilia Sfetcu; Lester Shulman; Annette Siedler; Ian Simms; Andreas Sing; Vitali Sintchenko; Gunilla Skoog; Robert Skov; Ramón Soriguer; Gianfranco Spiteri; Erin Staples; Cristina Stasi; Kim Steegen; Paola Stefanelli; Pawel Stefanoff; Marc Stegger; Chen Stein-Zamir; John Stenos; Alistair Story; Errol Strain; Franc Strle; Bertrand Sudre; Carl Suetens; Jonathan Suk; Sheena Sullivan; Edit Szegedi; Muhamed-Kheir Taha; Emi Takashita; Theresa Tam; Lara Tavoschi; Melanie M Taylor; Karin Tegmark-Wisell; Anders Tegnell; Daniel Thomas; Lelia Thornton; Karen Tiley; Elizabeth Torrone; Maria Elena Tosti; Mathieu Tourdjman; Katy Town; Sotirios Tsiodras; Nikki Turner; Bianca Törös; Bernhard Ultsch; Tim Uyeki; Palle Valentiner-Branth; Wim Van Bortel; Marita van de Laar; Kees. C. van den Wijngaard; Wim van der Hoek; Marianne van der Sande; Sylvie van der Werf; Marieke J. van der Werf; Gary van Domselaar; Johan Van Griesven; Albert Jan van Hoek; Esther van Kleef; Edward van Straten; Pieter van Thiel; Ivo van Walle; Carmen Varela Santos; Istvan Varkonyi; Alkiviadis Vatopoulos; Sophie Vaux; Irene Veldhuijzen; Harry Vennema; Giulietta Venturi; Line Vold; Gwenaël Vourc’h; Sabine Vygen; Anders Wallensten; Crystal Watson; Pierre Wattiau; Nina Weis; Don Weiss; Dirk Werber; Guido Werner; Therese Westrell; Ole Wichmann; Katarina Widgren; Annelies Wilder-Smith; Hendrik Wilking; Kumanan Wilson; Carl-Heinz Wirsing von König; Katja Wolthers; William WL Wong; Edwina Wright; Alban Ylli; Hans L Zaaijer; Katherina Zakikhany; Philipp Zanger; Gianluigi Zanusso; Peter Zarb; Gernot Zarfel; Herve Zeller; Ruth Zimmermann; Walter Zingg; Phillip Zucs; Milan Čižman; Barbara Šoba.

